# Hematological Adaptations to Prolonged Heat Acclimation in Endurance-Trained Males

**DOI:** 10.3389/fphys.2019.01379

**Published:** 2019-11-01

**Authors:** Laura Oberholzer, Christoph Siebenmann, C. Jacob Mikkelsen, Nicklas Junge, Jacob F. Piil, Nathan B. Morris, Jens P. Goetze, Anne-Kristine Meinild Lundby, Lars Nybo, Carsten Lundby

**Affiliations:** ^1^The Centre of Inflammation and Metabolism and the Centre for Physical Activity Research, Rigshospitalet, University of Copenhagen, Copenhagen, Denmark; ^2^Institute of Mountain Emergency Medicine, EURAC Research, Bolzano, Italy; ^3^Department of Nutrition, Exercise and Sport Sciences, University of Copenhagen, Copenhagen, Denmark; ^4^Department of Clinical Biochemistry, University of Copenhagen, Copenhagen, Denmark; ^5^Department of Biomedical Sciences, University of Copenhagen, Copenhagen, Denmark; ^6^Innland Norway University of Applied Sciences, Lillehammer, Norway

**Keywords:** hemoglobin mass, blood volume, critmeter, hematocrit, vasopressin, erythropoietin

## Abstract

Heat acclimation is associated with plasma volume (PV) expansion that occurs within the first week of exposure. However, prolonged effects on hemoglobin mass (Hb_mass_) are unclear as intervention periods in previous studies have not allowed sufficient time for erythropoiesis to manifest. Therefore, Hb_mass_, intravascular volumes, and blood volume (BV)-regulating hormones were assessed with 5½ weeks of exercise-heat acclimation (HEAT) or matched training in cold conditions (CON) in 21 male cyclists [(mean ± SD) age: 38 ± 9 years, body weight: 80.4 ± 7.9 kg, VO_2peak_: 59.1 ± 5.2 ml/min/kg]. HEAT (*n* = 12) consisted of 1 h cycling at 60% VO_2peak_ in 40**°**C for 5 days/week in addition to regular training, whereas CON (*n* = 9) trained exclusively in cold conditions (<15**°**C). Before and after the intervention, Hb_mass_ and intravascular volumes were assessed by carbon monoxide rebreathing, while reticulocyte count and BV-regulating hormones were measured before, after 2 weeks and post intervention. Total training volume during the intervention was similar (*p* = 0.282) between HEAT (509 ± 173 min/week) and CON (576 ± 143 min/week). PV increased (*p* = 0.004) in both groups, by 303 ± 345 ml in HEAT and 188 ± 286 ml in CON. There was also a main effect of time (*p* = 0.038) for Hb_mass_ with +34 ± 36 g in HEAT and +2 ± 33 g in CON and a tendency toward a higher increase in Hb_mass_ in HEAT compared to CON (time × group interaction: *p* = 0.061). The Hb_mass_ changes were weakly correlated to alterations in PV (*r* = 0.493, *p* = 0.023). Reticulocyte count and BV-regulating hormones remained unchanged for both groups. In conclusion, Hb_mass_ was slightly increased following prolonged training in the heat and although the mechanistic link remains to be revealed, the increase could represent a compensatory response in erythropoiesis secondary to PV expansion.

## Introduction

Natural heat acclimatization as well as laboratory-based heat acclimation translates into plasma volume (PV) expansion within the first few days of exposure (Périard et al., 2016). Longer intervention periods are typically required for the corresponding expansion in red blood cell volume (RBCV) and total hemoglobin mass (Hb_mass_) (Siebenmann et al., 2017b; Montero and Lundby, 2018) but are still desirable due to the potential for elevating arterial O_2_ delivery and improving endurance performance (Ekblom et al., 1972; Montero et al., 2015). Previous studies have, however, employed relatively short heat acclimation protocols leaving limited time for erythropoiesis to compensate for the hemodilution accompanying the initial PV expansion (Patterson et al., 2004; Keiser et al., 2015; McCleave et al., 2017; Rendell et al., 2017). Therefore, we tested whether exercise training in the heat, i.e., exercise-heat acclimation performed over a period of 5½ weeks, elicits higher Hb_mass_.

RBCV and Hb_mass_ expand in response to conventional endurance training (ET) which manifests after 4–6 weeks of ET in untrained individuals (Montero et al., 2015, 2017). In endurance athletes with high Hb_mass_, on the other hand, this effect is blunted throughout the season or after intense training periods (Gore et al., 1997; Prommer et al., 2008) and additional environmental or cardiovascular stressors may be required to prompt Hb_mass_ expansion further in such athletes. Therefore, hypoxic exposure or altitude training are strategies that are commonly employed by athletes, although their use is highly debated (Lundby and Robach, 2016; Bejder and Nordsborg, 2018). Prolonged exercise-heat acclimation is an alternative approach that potentially increases Hb_mass_ which however remains to be explored.

A potential mechanism underlying an expansion in Hb_mass_ may relate to the early PV expansion concomitant to exercise-heat acclimation as the reduced hematocrit, and thus arterial O_2_ content, triggers the release of erythropoietin (EPO) from the kidney (Adamson, 1968; Montero and Lundby, 2019). Indeed, the kidney has been proposed to act as a “critmeter,” regulating hematocrit by adjusting RBCV and PV mediated by EPO (Donnelly, 2001). Also, increased PV after 2 weeks of ET coincides with elevated EPO while RBCV remains unaffected which supports that a reduced hematocrit due to a sole expansion in PV may regulate erythropoiesis (Montero et al., 2017). It is also noteworthy that key PV-regulating hormones, e.g., vasopressin and angiotensin II exert direct effects on erythropoiesis (Engel and Pagel, 1995; Kim et al., 2014; Montero and Lundby, 2018). Thus, both an expansion in PV but also the changes in PV-regulating hormones could ultimately affect Hb_mass_. We therefore conducted the present study to test the hypothesis that exercise-heat acclimation for 5½ weeks would stimulate erythropoiesis and increase total Hb_mass_ in endurance-trained individuals and aimed at identifying some of the potential underlying hormonal and hematological mechanisms.

## Materials and Methods

The presented data were obtained as part of a large study exploring the effects of prolonged exercise-heat acclimation on performance and the underlying hematological mechanisms. For performance data, the reader is referred to the accompanying paper submitted in this issue (Mikkelsen et al., 2019, submitted). The study protocol was approved by the ethical committee of the Capital Region of Denmark (H-17036662) and conformed to the Declaration of Helsinki.

### Participants

Twenty-one healthy, endurance-trained, male cyclists provided oral and written consent for participation and were included in this study (Table 1). All participants conducted their regular cycling training during the preceding 3 months in cold temperatures outside (winter: <15**°**C) and were thus not heat acclimatized prior to commencement of the intervention.

**Table 1:** Participant characteristics at baseline.

	HEAT (*n* = 12)	CON (*n* = 9)
Age (years)	38.8 ± 8.9	37.7 ± 9.3
Body mass (kg)	80.2 ± 6.3	80.6 ± 9.5
Height (cm)	185 ± 3	184 ± 4
Body fat (%)	13.7 ± 4.0	14.7 ± 2.9
VO_2peak_ (L/min)	4.8 ± 0.4	4.6 ± 0.4
VO_2peak_ (ml/min/kg)	60.0 ± 5.1	57.9 ± 5.1
Training volume pre (min/week)	417 ± 105	499 ± 164
Training volume during (min/week)	509 ± 173	576 ± 143
Training volume > 80% HR_max_ pre (min/week)	102 ± 71	102 ± 55
Training volume > 80% HR_max_ during (min/week)	157 ± 90	122 ± 57

*HR_max_, maximal heart rate; VO_2peak_, peak oxygen uptake*.

*Pre refers to before the intervention. Data are presented as mean ± SD*.

### Study Design

Participants first underwent baseline testing consisting of blood sampling and determination of body composition, peak oxygen uptake (VO_2peak_), Hb_mass_, and intravascular volumes. After baseline measurements, participants were age- and VO_2peak_-matched into two groups which were thereafter randomly assigned as the exercise-heat acclimation (HEAT, *n* = 12) or the control (CON, *n* = 9) group. Participants then completed the 5½-week intervention period, where after blood sampling and determination of Hb_mass_ and intravascular volumes was repeated. In addition, blood sampling was conducted after 2 weeks into the intervention period prior to an exercise training session.

### Intervention

HEAT conducted 1 h of cycling in a climatic chamber on 5 weekly occasions for 5½ weeks (28 ± 2 sessions in total). Temperature in the climatic chamber corresponded to 35**°**C in the first week and was augmented by 1**°**C each week (relative humidity of 30 ± 8%). This gradual increment in temperature provided a constant adaptation stimulus and resulted in a rectal temperature of >38.5**°**C after 35 ± 8 min of training during all training sessions. Airflow was provided by a fan only if the participant could not complete the training otherwise and participants were allowed to drink warm water *ad libitum* during the training. CON maintained their regular outdoor training (<15**°**C) but reported to the laboratory once a week and cycled in cold conditions (<15**°**C) to maintain familiarization to stationary cycling. All training sessions in the laboratory, i.e., in the climatic chamber for HEAT, consisted of cycling at 60% VO_2peak_ as determined in cold conditions (~15**°**C) and were conducted on the participants’ personal bikes using a stationary Tacx-trainer device (Tacx Neo Smart T2800; Tacx, Netherlands) and associated software (Tacx Trainer software 4; Tacx, Netherlands). Participants in both groups completed a training log to quantify their training volume and intensity (assessed by heart rate) 2 weeks prior to the intervention and 2 weeks into the intervention. Participants were instructed to maintain their training routine throughout the intervention but to subtract the training hours performed in the laboratory from their regular training. This resulted in similar training volumes between HEAT and CON.

### Measurements

#### Body Composition

Baseline body mass and fat percentage were assessed by bioimpedance (InBody 270; InBody, Denmark).

#### Peak Oxygen Uptake

An incremental exercise test was performed to determine VO_2peak_. The test was conducted on the participants’ personal bikes, which were installed on a stationary Tacx-trainer device (Tacx Neo Smart T2800; Tacx, Netherlands). Following a 10 min warm up with 5 min at 100 W and 5 min at 175 W (80 RPM), workload was increased by 25 W/min until exhaustion. VO_2_ and VCO_2_ were obtained by breath-by-breath recordings (Jaeger Oxycon Pro; Viasys Healthcare, Germany). The gas analyzers and the flowmeter were calibrated before each test. A plateau in VO_2_ despite increased workload and/or attainment of a respiratory exchange ratio (RER) ≥ 1.15 served as test validation criteria. VO_2peak_ was defined as the highest observed value over a 30s-period.

#### Hemoglobin Mass and Intravascular Volumes

Hb_mass_ and intravascular volumes were assessed using the carbon monoxide (CO) rebreathing technique (Siebenmann et al., 2017a). For some of the participants (*n* = 11), an automated version of the CO rebreathing (OpCO; Detalo Health, Denmark) was used. The same method (manual/automated) was applied for intra-individual pre-post comparisons and the distribution of which technique was used was random among HEAT (*n* = 7) and CON (*n* = 4). The procedure was as follows: the participant rested for 20 min in the supine position before each measurement. During this time, the participant drank 500 ml of water and an 18-G venous catheter was placed into an antecubital vein. The participant was then connected to a breathing circuit and breathed 100% O_2_ for 4 min. 2 ml of blood were sampled and analyzed immediately in quadruplicates for (1) percent carboxyhemoglobin (%HbCO) and hemoglobin concentration ([Hb]) (ABL835; Radiometer, Denmark) and (2) hematocrit with the microcentrifuge method (4 min at 13,500 RPM). Subsequently, the participant was switched by a sliding valve to a O_2_-filled rebreathing circuit and a bolus of 1.5 ml/kg body weight of 99.997% chemically pure CO (CO N47; Strandmøllen, Denmark) was administered to the rebreathing circuit. O_2_ was supplied into this circuit on a demand basis. The participant rebreathed the O_2_-CO gas mixture for 10 min. A second blood sample was obtained after 10 min of CO rebreathing and analyzed in quadruplicates for %HbCO. The remaining CO volume in the rebreathing circuit was determined as previously specified (Siebenmann et al., 2017a) and was subtracted from the applied CO dose. For the calculation of Hb_mass_, the absorbed CO dose and the changes in %HbCO from before to after rebreathing were used. Total blood volume (BV), RBCV, and PV were then derived from Hb_mass_, [Hb], and hematocrit (Burge and Skinner, 1995).

#### Blood Sampling and Analyses

Venous blood was collected in EDTA-coated tubes for analyses of [Hb] and reticulocyte count (Sysmex XN; Sysmex Europe, Germany) on whole blood. Furthermore, 2 ml of blood was collected in a heparinized syringe (PICO50; Radiometer, Denmark) to analyze blood electrolyte concentration with an automated hemoximeter (ABL835; Radiometer, Denmark). A third blood sample was obtained in a sodium heparin-coated vacutainer. After centrifugation, plasma was collected and stored at −80°C until further analysis. Plasma EPO was determined with an ELISA kit (Human Erythropoietin Quantikine IVD ELISA Kit; R&D Systems, USA) with an intra-assay coefficient of variation (CV) of 2.8–5.2% and inter-assay CV of <1%. Plasma protein and albumin concentrations were measured with an automated analyzer (Cobas 8,000, c702 modul; Roche, Germany) with an intra- and inter-assay CV of <5%. Total protein and albumin were calculated by multiplying the respective concentrations with PV. Plasma copeptin as a more stable proxy for vasopressin was determined using an automated immunofluorescent assay (Thermo Fisher Scientific BRAHMS; Germany) (Alehagen et al., 2011), while pro-ANP was measured with a mid-regional assay on a Kryptor Plus platform (Thermo Fisher Scientific BRAHMS; Germany) (Hunter et al., 2011), both with intra- and inter-assay CV of <6.5%.

#### Statistical Analyses

All statistical analyses were performed using SPSS 22 (IBM SPSS Statistics, USA). Figures were made using GraphPad Prism 8.0.0 (GraphPad Software; USA). Power calculations before the onset of the study estimated that a sample size of *n* ≥ 9 in each group would allow detecting a meaningful change in Hb_mass_. Prior to analyses, data were evaluated for normality and equal variance and were log-transformed if required. Independent t-test was applied to assess differences in training volume between HEAT and CON. The influence of HEAT on the effects of ET on Hb_mass_, intravascular volumes and hematological parameters was assessed with a two-way repeated measures ANOVA. Main effects of time (pre-post ET) as within-subject factor and of group (HEAT-CON) as between-subject factor were determined along with the corresponding interactions. In addition, Pearson’s correlation coefficient was computed to assess associations between hematological parameters. Data are expressed as means ± standard deviation (SD). *p* <0.05 was considered statistically significant.

## Results

Heat acclimation in HEAT was verified by improved exercise tolerance in the heat and lowered sweat sodium concentration, while no signs of heat acclimation were observed for CON [see Mikkelsen et al. (2019), submitted, for details].

### Hemoglobin Mass and Intravascular Volumes

**Figure 1:**
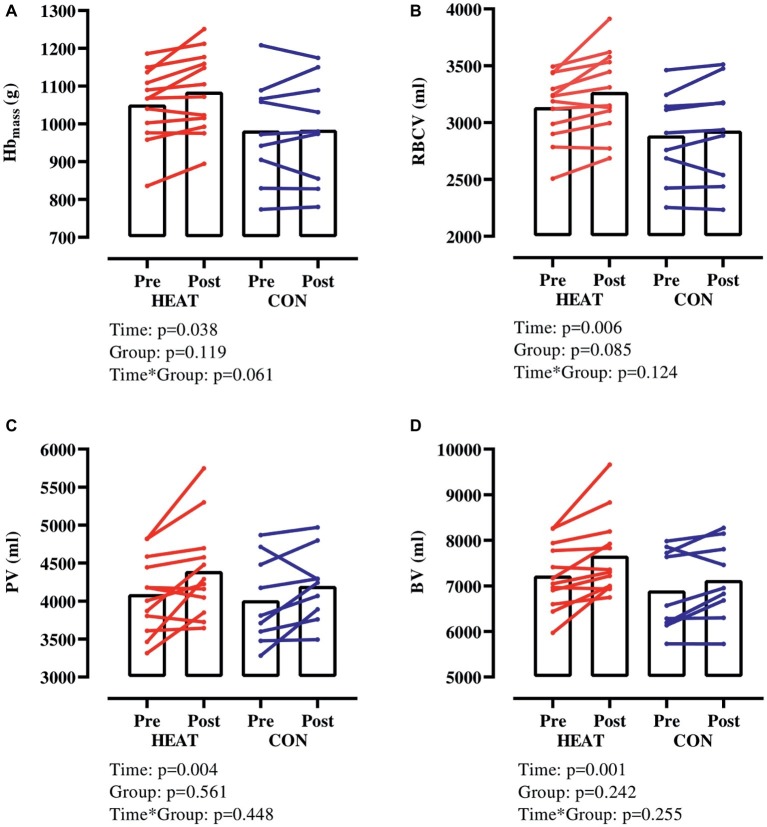
Hb_mass_ and intravascular volumes with exercise-heat acclimation (HEAT) or matched control training (CON). **(A)** hemoglobin mass (Hb_mass_), **(B)** red blood cell volume (RBCV), **(C)** plasma volume (PV), **(D)** blood volume (BV).

Hb_mass_ increased in both groups (*p* = 0.038) but this increase tended (*p* = 0.061) to be larger in HEAT (+3.2 ± 3.3% from 1,052 ± 97 to 1,085 ± 108 g) than in CON (+0.2 ± 3.2% from 983 ± 137 to 985 ± 141 g) (Figure 1A). RBCV increased in both groups (*p* = 0.006) from 3,136 ± 295 to 3,270 ± 364 ml (+4.2 ± 4.2%) in HEAT and from 2,888 ± 395 to 2,929 ± 453 ml (+1.3 ± 3.3%) in CON (Figure 1B). Also, PV increased in both groups (*p* = 0.004) from 4,091 ± 506 to 4,394 ± 626 ml (+7.6 ± 8.7%) in HEAT and from 4,012 ± 569 to 4,200 ± 471 ml (+5.3 ± 7.5%) in CON (Figure 1C). As a result of the elevated RBCV and PV, BV was expanded (*p* = 0.001) from 7,227 ± 725 to 7,664 ± 876 ml (+6.1 ± 5.9%) in HEAT and from 6,900 ± 884 to 7,130 ± 858 ml (3.5 ± 4.6%) in CON (Figure 1D). There was no time × group interaction for RBCV, PV or BV.

### General Hematological Characteristics and Plasma Hormones

Hematocrit, [Hb] and reticulocyte count remained unaffected throughout the intervention in both groups (Table 2). There was an effect of time for mean corpuscular hemoglobin concentration (*p* = 0.015) and for plasma albumin (*p* = 0.014) and protein concentration (*p* = 0.028), however, no effect of group or interaction of time ´ group were detected. Likewise, total albumin and protein content increased in both groups (*p* = 0.004 and *p* < 0.001, respectively). Plasma EPO, pro-ANP and copeptin remained unchanged. Furthermore, blood sodium, chloride, calcium and potassium concentrations were unchanged in both groups.

**Table 2:** Hematological characteristics and plasma hormone concentrations.

	HEAT			CON		
	Pre	Mid	Post	Pre	Mid	Post
[Hb] (g/dl)	14.5 ± 0.9	14.6 ± 1.0	14.3 ± 1.0	14.0 ± 1.0	14.4 ± 0.9	14.2 ± 0.8
Hematocrit (%)	43.5 ± 2.5	—	42.8 ± 3.2	41.9 ± 2.8	—	41.0 ± 2.6
Reticulocytes (10^9^/L)	52.0 ± 8.9	59.3 ± 14.8	54.8 ± 16.2	55.3 ± 14.1	60.4 ± 12.8	54.8 ± 16.3
MCHC (g/dl)	33.4 ± 0.9	—	34.1 ± 2.0	33.4 ± 1.3	—	35.3 ± 2.5
Plasma proteins (g/L)	73.2 ± 3.9	75.7 ± 5.3	74.6 ± 2.6	74.4 ± 3.4	78.4 ± 4.9	76.3 ± 2.7
TCP (g)	298 ± 29	—	327 ± 46	297 ± 34	—	321 ± 38
Albumin (g/L)	42.8 ± 2.1	44.5 ± 2.8	42.5 ± 2.0	43.3 ± 3.0	45.0 ± 5.2	43.8 ± 2.9
Total albumin (g)	175 ± 20	—	187 ± 27	173 ± 19	—	184 ± 20
Copeptin (pmol/L)	68.3 ± 26.2	65.0 ± 18.1	78.6 ± 27.4	59.7 ± 24.6	58.4 ± 27.1	65.2 ± 24.7
Pro-ANP (pmol/L)	5.71 ± 2.29	6.20 ± 3.24	5.57 ± 2.45	4.57 ± 1.72	4.30 ± 1.13	5.00 ± 1.82
EPO (mIU/ml)	9.3 ± 2.9	8.7 ± 1.7	9.4 ± 2.2	9.7 ± 2.3	9.4 ± 3.4	9.3 ± 1.8

*EPO, erythropoietin; [Hb], hemoglobin concentration; MCHC, mean corpuscular hemoglobin concentration; TCP, total content of protein; Pro-ANP, pro-atrial natriuretic peptide*.

*Before (Pre), after 2 weeks (Mid) and after the intervention (Post). Results represent mean ± SD*.

### Correlations

**Figure 2:**
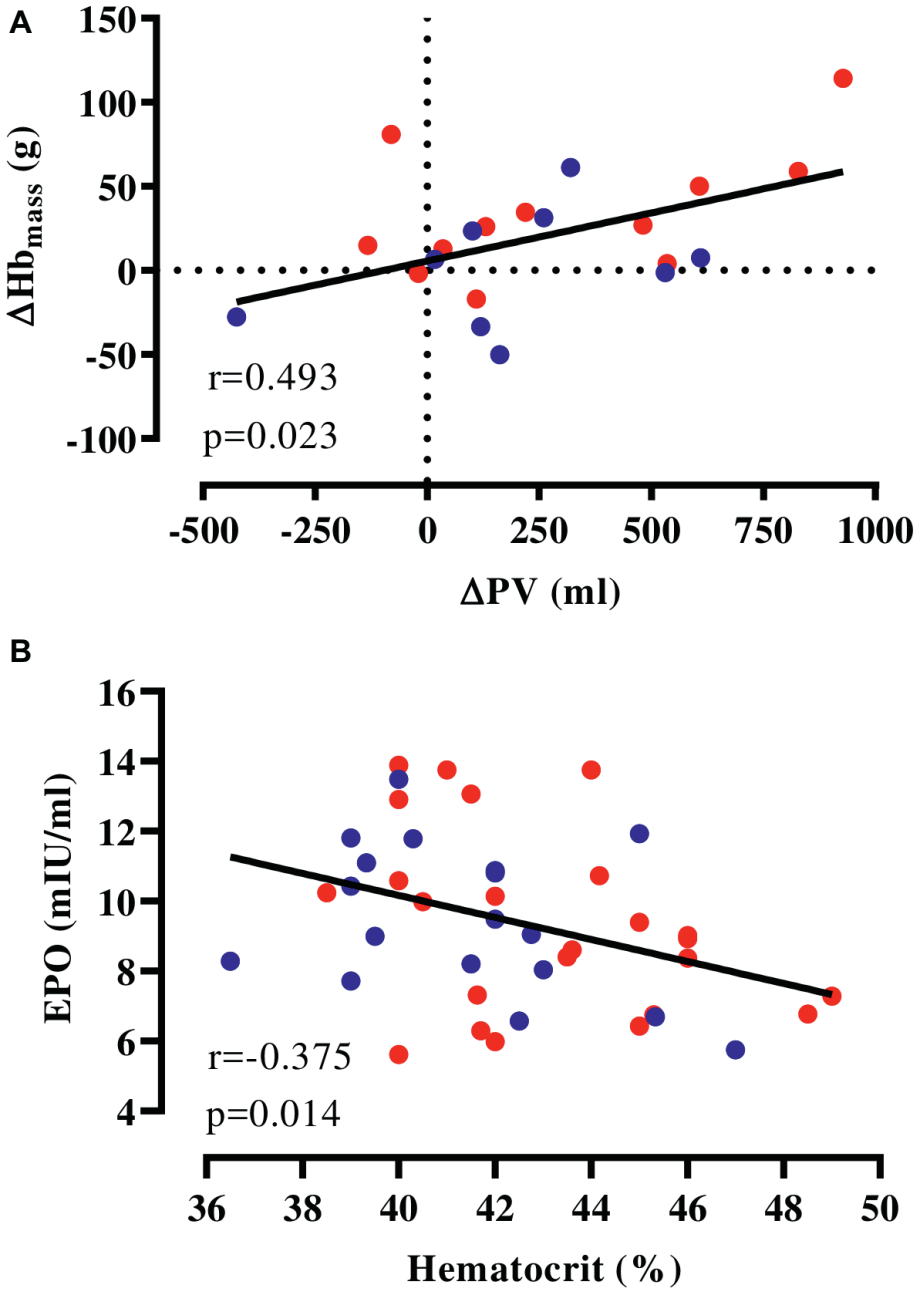
Correlation of hematological parameters. **(A)** Changes in Hb_mass_ and PV with the intervention (HEAT: red, CON: blue), **(B)** absolute EPO and hematocrit (values before and after the intervention pooled). EPO, erythropoietin; Hb_mass,_ hemoglobin mass; PV, plasma volume.

We pooled HEAT and CON to examine whether the expansion in PV is correlated to accentuated erythropoiesis and found that changes in PV were weakly associated with altered Hb_mass_ in response to the intervention (Figure 2A). Furthermore, hematocrit determined before and after the intervention was negatively associated with plasma EPO at these time points (Figure 2B) and similarly, there was a tendency toward a negative association (*r* = −0.416, *p* = 0.076) between [Hb] and EPO determined at 2 weeks into the intervention. However, no association of copeptin and pro-ANP with EPO was detected at any time point.

## Discussion

The present study provides a detailed picture of the hematological adaptations to prolonged exercise-heat acclimation and we report a 3% increase in Hb_mass_ following heat acclimation corresponding to a change of +34 g (range: −17 to 114 g) for HEAT compared to +2 g (range: −50 to 61 g) for CON. This observation is in agreement with our hypothesis, although we only observed a tendency toward a higher increase in Hb_mass_ after exercise-heat acclimation compared to matched training in cold conditions. The Hb_mass_ expansion was weakly correlated to the overall PV change, indicating that the PV expansion is accompanied by an elevation of total Hb_mass_. Hence, we suggest that in endurance-trained individuals with high Hb_mass_, heat imposed on ET may trigger a further erythropoietic stimulus, leading to additional Hb_mass_ expansion.

Studies on the adaptation of Hb_mass_ to heat exposure are rare and equivocal. Although, some report unchanged Hb_mass_ in response to 10 or 21 days of exercise-heat acclimation (McCleave et al., 2017; Rendell et al., 2017), we hypothesized these training durations were insufficient to elicit increased erythropoiesis. A reason for this hypothesis was that higher RBCV and Hb_mass_ is only detected after >4 weeks of conventional ET in untrained individuals (Montero et al., 2017). Indeed, in the present study, 5½ weeks of exercise-heat acclimation elicited a slight expansion of 34 g, whereas Hb_mass_ in CON remained similar with +2 g. This ∼3% Hb_mass_ expansion in HEAT was greater than the typical error of measurement of the CO rebreathing we observe in our laboratory when using the manual method (Siebenmann et al., 2015, 2017a) and when using the automated version (Fagoni et al., 2018). Higher Hb_mass_ has also previously been reported ∼3½ weeks after the initiation of an exercise-heat acclimatization period (Karlsen et al., 2015). Opposite to exercise-heat acclimation as in the present study, participants were residing and training in a natural hot environment thus heat exposure time was substantially longer. However, similar exercise-heat acclimatization has also resulted in unaltered Hb_mass_ (Gore et al., 1997). Hence, whether exercise-heat acclimatization manifests in erythropoietic adaptation remains controversial. Overall, applying exercise-heat acclimation, i.e. laboratory-based intermittent heat exposure appears to trigger an erythropoietic response and may be easier to implement, as it does not involve traveling to hot areas and allows furthermore to carefully control for exposure temperature and humidity.

It is recognized that heat exposure and undergoing exercise-heat acclimation or acclimatization results in an expansion of PV between 3 and 27% within the first days of exposure (Périard et al., 2016). We observed that exercise-heat acclimation may furthermore pose an erythropoietic stimulus. In fact, the higher Hb_mass_ may be a consequence of the exercise-heat acclimation-induced PV expansion (Montero and Lundby, 2018). The mechanistic basis for this was introduced with the concept of the kidney functioning as a “critmeter” that controls hematocrit by adjusting PV and RBCV, and thus stabilizes arterial O_2_ content (Donnelly, 2001). The mediating hormone is the glycoprotein EPO that is released upon renal tissue hypoxia resulting from hemodilution and promotes the production of red blood cells in the hematopoietic bone marrow (Jelkmann, 2011). Indeed, it is observed that the rise in EPO coincides with the expansion in PV after 2 weeks of ET (Montero et al., 2017). In the present study, we found that alterations in Hb_mass_ were weakly correlated to PV changes when participants from HEAT and CON were included in the analysis. Furthermore, participants with low hematocrit possessed higher plasma EPO as previously reported for anemic individuals (Erslev, 1991). Thus, accumulating evidence, including our correlational data, points toward PV fluctuations being a driver of erythropoiesis. It needs to be highlighted, however, that the tendency in higher Hb_mass_ in HEAT was not reflected in any changes in plasma EPO or other BV-regulating hormones measured after 2 weeks and by the end of the intervention and the above hypothesis is only supported by the correlational analyses. Moreover, the [Hb] was unchanged after 2 weeks into the intervention period, indicating either normalized PV at this time point or partial Hb_mass_ expansion already compensating for elevated PV. While the latter appears unlikely (Keiser et al., 2015; Rendell et al., 2017), there is some evidence pointing toward only transient effects of exercise-heat acclimatization on PV (Wyndham et al., 1968). Since CO rebreathing was omitted during the intervention period, we cannot conclude on the precise time course of Hb_mass_ and intravascular volume adaptations to exercise-heat acclimation. In addition, determination of the PV-regulating hormones pro-ANP and copeptin, a proxy measure of vasopressin, did not reveal any alterations.

The strong association between endurance performance and Hb_mass_ implies that strategies to stimulate and induce an overall increase in Hb_mass_ are commonly applied by endurance athletes (Gore et al., 1997). A classic procedure is altitude training or “live high-train low”, where the hypoxia-induced augmented RBCV is observed to enhance performance (Stray-Gundersen and Levine, 2008), although more recent evidence questions this approach (Bejder and Nordsborg, 2018; Robach et al., 2018). Nonetheless, data showing Hb_mass_ expansion with altitude training in individuals with a similarly high Hb_mass_ as in the present study, report an increase of 5–6% (Robach and Lundby, 2012), which is slightly higher than the +3% observed in the current study with prolonged exercise-heat acclimation. Notably, hypoxia leads to an early contraction in PV (Siebenmann et al., 2017b), whereas exercise-heat acclimation is a training approach that circumvents this reduction in PV.

Eventually, the question arises as to whether the trend in higher Hb_mass_ translated into better endurance performance. It is known that the infusion of packed red blood cells leads to improved VO_2peak_ consequent of increased O_2_ transport capacity and facilitated cardiac output (Ekblom et al., 1972). The autologous transfusion of ~135 ml red blood cells is furthermore sufficient to improve time trial performance by ~5% in well-trained men (Bejder et al., 2019). Considering that participants in HEAT in the present study had elevated RBCV by 134 ± 140 ml, some participants may indeed have benefitted from a performance effect in cold conditions. However, while there was an improved time trial performance in HEAT in cold conditions, the same was observed for CON and the intervention did not affect VO_2peak_ [Mikkelsen et al. (2019), submitted in this issue]. Yet, it has been suggested that VO_2peak_ is elevated by ~4 ml/min for each1g rise in Hb_mass_ (Schmidt and Prommer, 2010), which hypothetically would correspond to a mean increase in VO_2peak_ of ~1.75 ml/min/kg (+3%) in HEAT. While this slight increment is of relevance for competing athletes, it is likely that our VO_2_ measurement was not sufficiently sensitive to detect this difference (Carter and Jeukendrup, 2002). Taken together, even though Hb_mass_ and thus O_2_ transport capacity tended to be higher in HEAT than in CON, this did not manifest in better performance in the cold. Nonetheless, it is worthwhile investigating whether exercise-heat acclimation for even longer periods results in a gradual Hb_mass_ expansion and whether that may ultimately improve endurance performance in the cold.

We acknowledge some limitations to our study. First, we only observed a tendency toward a modifying effect of exercise-heat acclimation on Hb_mass_. This tendency is likely related to the variation in the two groups and therefore limited statistical power may hinder us from drawing definite conclusions. Nevertheless, the time × group interaction was borderline significant (*p* = 0.061) and the inclusion of a carefully matched control group (VO_2peak_, age, and training volume) is considered as a major strength of the study design. Secondly, we were not able to pinpoint the time course of erythropoietic adaptation to exercise-heat acclimation, as reticulocyte count and EPO remained unaltered at 2 weeks of exercise-heat acclimation. This is in contrast to conventional ET, where EPO peaks after 2 weeks and thereafter returns to baseline (Montero et al., 2017). However, at altitude, a steep rise in EPO is detected already after 24 h of exposure whereafter it normalizes (Lundby et al., 2014) and it thus may be that the erythropoietic stimulus in the present study occurred earlier. Thirdly, we hypothesized that the expansion in PV is a mechanism underlying the higher Hb_mass_. However, we only revealed a weak correlation between alterations in PV and Hb_mass_ and we can thus only speculate on a potential association of the PV expansion with the higher Hb_mass_. Accordingly, there is need for further experimental verification in humans (Montero and Lundby, 2018). Ultimately, even though participants in the present study were endurance-trained, they did not reach the very high Hb_mass_ values of professional endurance athletes (Jelkmann and Lundby, 2011). Given that it appears challenging to augment erythropoiesis in athletes with high initial Hb_mass_ (Robach and Lundby, 2012), it remains to be examined whether prolonged exercise-heat acclimation in professional endurance athletes is feasible and beneficial for Hb_mass_ expansion.

In summary, when endurance-trained individuals were exposed to environmental stress, i.e., heat, during a substantial part of their weekly training, Hb_mass_ tended to be more expanded than with conventional ET. The mechanisms triggering the response remain to be revealed but could involve a compensatory response in erythropoiesis secondary to PV expansion as the higher Hb_mass_ was correlated to the expansion in PV although EPO and BV-regulating hormones remained unchanged.

## Data Availability Statement

All datasets generated for this study are included in the article/supplementary files. Some data can be found in the accompanying article (Article DOI: 10.3389/fphys.2019.01372).

## Ethics Statement

The studies involving human participants were reviewed and approved by the Ethics Committee of the Capital Region of Denmark, protocol no.: H-17036662. The patients/participants provided their written informed consent to participate in this study.

## Disclosures

The license for the product Tacx Trainer device and software was obtained from the copyright holders.

## Author Contributions

LN and CL contributed in conception and design of research. LO, CS, CM, NJ, JP, JG, and A-KM performed experiments. LO and CS analyzed data. LO, CS, NM, LN, and CL interpreted results of experiments and drafted the manuscript. LO prepared figures. LO, CS, CM, NJ, JP, NM, JG, A-KM, LN, and CL approved the final version of manuscript and edited and revised the manuscript.

### Conflict of Interest

The authors declare that the research was conducted in the absence of any commercial or financial relationships that could be construed as a potential conflict of interest.
